# *Transitions*: Novel Study Methods to Understand Early HIV Risk Among Adolescent Girls and Young Women in Mombasa, Kenya, and Dnipro, Ukraine

**DOI:** 10.3389/frph.2020.00007

**Published:** 2020-09-10

**Authors:** Eve Cheuk, Sharmistha Mishra, Olga Balakireva, Helgar Musyoki, Shajy Isac, Daria Pavlova, Parinita Bhattacharjee, Robert Lorway, Michael Pickles, Huiting Ma, Peter Gichangi, Paul Sandstrom, Lyle R. McKinnon, Lisa Lazarus, Stephen Moses, James Blanchard, Marissa Becker

**Affiliations:** ^1^Centre for Global Public Health, Department of Community Health Sciences, Rady Faculty of Health Sciences, University of Manitoba, Winnipeg, MB, Canada; ^2^MAP Centre for Urban Health Solutions, Li Ka Shing Knowledge Institute, St. Michael's Hospital, Toronto, ON, Canada; ^3^Department of Medicine, University of Toronto, Toronto, ON, Canada; ^4^Institute of Medical Sciences, University of Toronto, Toronto, ON, Canada; ^5^Institute of Health Policy, Management and Evaluation, University of Toronto, Toronto, ON, Canada; ^6^Institute for Economics and Forecasting, Ukrainian National Academy of Sciences, Kyiv, Ukraine; ^7^Ukrainian Institute for Social Research After Oleksandr Yaremenko, Kyiv, Ukraine; ^8^National AIDS and STI Control Programme, Ministry of Health, Nairobi, Kenya; ^9^India Health Action Trust, New Delhi, India; ^10^Imperial College London, London, United Kingdom; ^11^International Centre for Reproductive Health Kenya, Mombasa, Kenya; ^12^Technical University of Mombasa, Mombasa, Kenya; ^13^National HIV and Retrovirology Laboratories, Public Health Agency of Canada, Winnipeg, MB, Canada; ^14^Department of Medical Microbiology and Infectious Diseases, Rady Faculty of Health Sciences, University of Manitoba, Winnipeg, MB, Canada

**Keywords:** female sex workers, adolescent girls and young women, HIV, prevention, Kenya, Ukraine

## Abstract

*Transitions* aims to understand the human immunodeficiency virus (HIV) risk at critical transition points in the sexual life course of adolescent girls and young women (AGYW) who engage in casual sex, transactional sex, and sex work. In this article, we present the *Transitions* study methods. The *Transitions* study has the following objectives: (1) to describe how the characteristics and length of the transition period and access gap vary across two epidemiological contexts (Mombasa, Kenya, and Dnipro, Ukraine); (2) to understand how the risk of HIV varies by length and characteristics of the transition period and access gap across epidemiologic contexts; and (3) to assess the extent to which HIV infections acquired during the transition period and access gap could mitigate the population-level impact of focused interventions for female sex workers and explore the potential marginal benefit of expanding programs to reach AGYW during the transition period and access gap. Cross-sectional biobehavioral data were collected from young women aged 14 to 24 years who were recruited from locations in Mombasa County, Kenya, and Dnipro, Ukraine, where sex work took place. Data are available for 1,299 Kenyan and 1,818 Ukrainian participants. The survey addressed the following areas: timing of transition events (first sex, first exchange of sex for money or other resources, self-identification as sex workers, entry into formal sex work, access to prevention program services); sexual behaviors (condom use, anal sex, sex under the influence of drugs or alcohol); partnerships (regular and first-time clients, regular and first-time transactional sex partners, and husbands and boyfriends); alcohol use; injection and non-injection illicit drug use; experience of violence; access to HIV prevention and treatment program; testing for sexually transmitted and blood-borne infections and HIV; and reproductive health (pregnancies, abortions, contraceptives). HIV and hepatitis C virus prevalence data were based on rapid test results. Mathematical modeling will be used to generate projections of onward HIV transmission at specific transition points in the sexual life course of AGYW. Taken together, these data form a novel data resource providing comprehensive behavioral, structural, and biological data on a high-risk group of AGYW in two distinct sociocultural and epidemiologic contexts.

## Introduction

Across epidemic contexts, female sex workers (FSWs) and adolescent girls and young women (AGYW) experience a disproportionate burden of human immunodeficiency virus (HIV) (1)^1^. Globally, FSWs are 13 times more likely to be infected with HIV compared to females of reproductive age (1). In 2017, of the estimated 760,000 new HIV infections in adult women worldwide, 44.7% were among AGYW (aged 15–24 years) ^1^. AGYW accounted for 61.5 and 58.6% of persons aged 15 to 24 years who were living, and newly infected, with HIV in 2017, respectively ^1^.

Traditional programs tailored for FSWs do not officially provide services for AGYW engaged in sex work (SW) since the legal status of some AGYW as minors impedes their access to health services without parental consent (2, 3). Furthermore, HIV prevention interventions tailored for FSWs only reach women after they self-identify as sex workers. Yet, HIV prevalence data by age and duration in SW from Kenya suggest that women may be at increased risk for HIV prior to, and within, very early stages of SW (4, 5).

**Figure 1:**
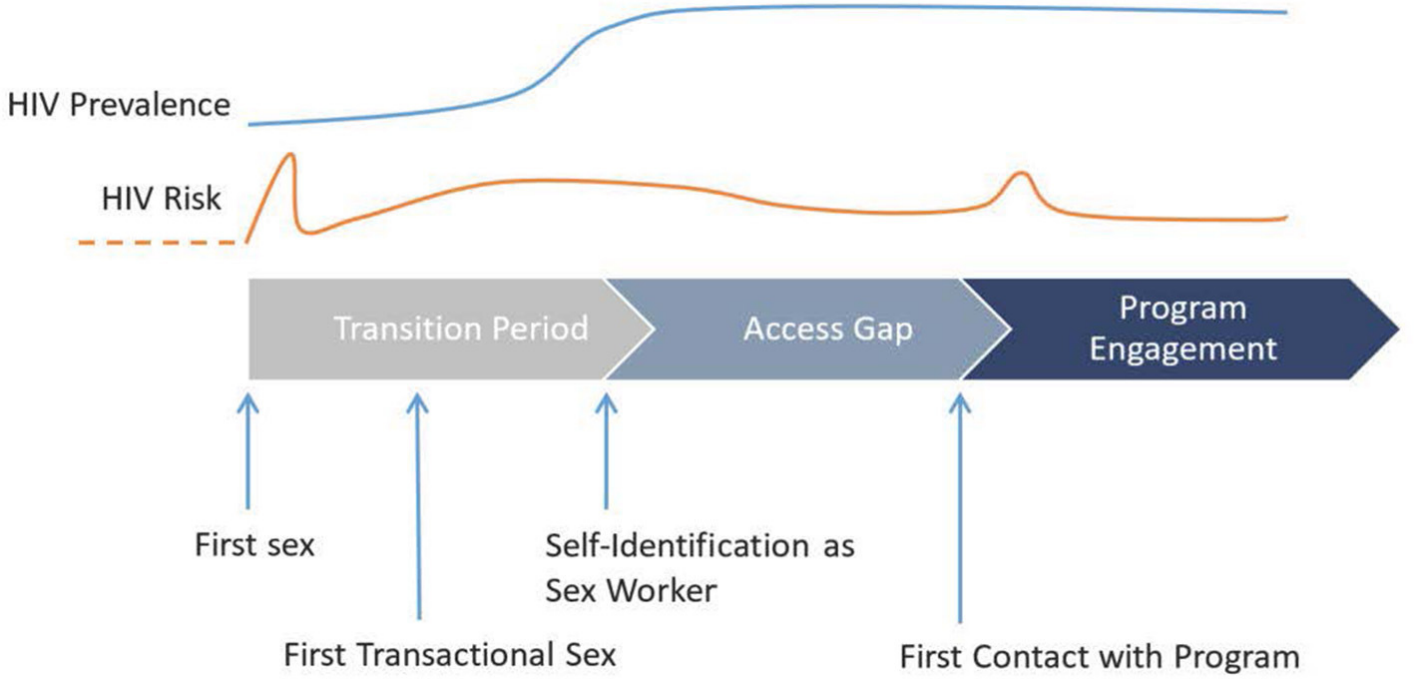
Schematic representation of the central concept of the *Transitions* study, depicting key events of a simplified linear sexual life course of young women in relation to HIV risk and prevalence and engagement with HIV prevention programming, from first sex to self-identification as sex workers (i.e., transition period) and from self-identification as sex workers to first contact with HIV programming (i.e., access gap).

The *Transitions* study was designed to measure the prevalence and patterns of HIV risk, vulnerabilities, and infection and to generate model-based projections of onward HIV transmission at specific transition points in the sexual life course of AGYW, including young FSWs, in two epidemic contexts (Kenya and Ukraine) (6, 7). Specifically, the study was designed to characterize the factors that influence HIV risk during the *transition period* (from first sex until self-declared entry into SW) and during the *access gap* (after entry into formal SW but before HIV prevention program engagement) among young women who enter SW (Figure 1) and to examine the potential role of early HIV risk in epidemic control. In this article, we present the *Transitions* study methods.

## Methods

### Study Design and Objectives

*Transitions* is a cross-sectional biobehavioral survey with the following objectives:

(1) to describe how the characteristics and length of the transition period and access gap vary across two epidemiological contexts (Mombasa, Kenya, and Dnipro, Ukraine);
(2) to understand how the risk of HIV varies by length and characteristics of the transition period and access gap across epidemiologic contexts; and
(3) to assess the extent to which HIV infections acquired during the transition period and access gap could mitigate the population-level impact of focused interventions for FSWs and explore the potential marginal benefit of expanding programs to reach AGYW during the transition period and access gap.

### Study Setting and Study Population

*Transitions* was implemented in Mombasa County, Kenya, and the city of Dnipro, Ukraine, in 2015. These two settings were chosen for their distinct sociocultural and epidemiological settings (Table 1).

**Table 1:** Characteristics of *Transitions* study sites—(A) Mombasa County, Kenya, and (B) City of Dnipro, Ukraine.

**(A) Mombasa County, Kenya**		
Population size (2015)	General population	1,179,929
	Females of reproductive age (14–44 years)	290,263
	Females aged 15–24 years	127,750
HIV prevalence	National (KAIS 2012)	
	All (KAIS 2012)	5.6%
	Females (KAIS 2012)	6.9%
	Males (KAIS 2012)	4.2%
	Female sex workers (IBBS 2010)	29.3%
	Mombasa County	
	All (KAIS 2012)	7.4%
	Females (KAIS 2012)	10.5%
	Males (KAIS 2012)	4.5%
	Females aged 15–19 years (MCASP)	6.0%
	Females aged 20–24 years (MCASP)	10.0%
	Female sex workers	Not known
Sex work hotspot typology	Public place	
	Street	
	Bar/nightclub/casino/hotel (with rooms)	
	Bar/restaurant/café (without rooms)	
	Guesthouse/lodge (without bars)	
	Sex den/brothel	
	Local brew den	
	Other (including home-based, massage parlors/saunas, video dens, and truck stops)	
**(B) Dnipro, Ukraine**		
Population size (2018)	General population	990,381
	Females of reproductive age (14–44 years)	207,478
	Females aged 15–24 years	46,797
HIV prevalence	National	
	All (2017)	1.4%
	Females	—
	Males	—
	Female sex workers (IBBS 2015)	7.0%
	City of Dnipro	
	All	0.7%
	Females	—
	Males	—
	Females aged 15–19 years	—
	Females aged 20–24 years	—
	Female sex workers (HIV Informational Bulletin No49, 2018)	2.5%
Sex work hotspot typology	Brothel	
	Open area (e.g., street, park etc.)	
	Highway, trick stop	
	Entertainment venue (e.g., night club, casino, discotheque, etc.)	
	Café, restaurant, bar	
	Massage salon, sauna, beauty parlor	
	Art club, strip bar	
	Hotel, motel	
	Apartment	
	Other (e.g., dormitories, etc.)	

#### Mombasa

Mombasa city is an important tourism center and major port that handles millions of tons of cargo annually. In 2015, Mombasa had a projected population of 1,179,929, including 290,263 females of reproductive age (14–44 years) and 127,750 AGYW (15–24 years) (8). The HIV prevalence in Mombasa County was 7.4%, (8) compared to a national prevalence of 5.6% (9). The HIV prevalence among all females was 10.5%, and 6% among those aged 15 to 19 years and 10% among those aged 20 to 24 years (8). The HIV prevalence among FSWs Mombasa was unknown (8).

#### Dnipro

Dnipro is an industrial city in eastern Ukraine. In 2018, Dnipro had a population of 990,381, including 207,478 females of reproductive age (14–44 years) and 46,797AGYW (15–24 years) (10). The HIV prevalence in Dnipro was 0.7%, compared to a national prevalence of 1.4%. The HIV prevalence among FSWs Dnipro was 2.5% (11).

The eligibility criteria for participation included *cis*-females who:

1) were 14 to 24 years of age;
2) had ever had vaginal and/or anal sex; and
3) were congregating at SW “hotspots” at the time of recruitment.

A hotspot is an indoor or outdoor location where FSWs congregate to meet clients. The hotspot typology differs between Mombasa and Dnipro (Table 1).

In this study, participants were categorized into one of three study groups—SW, transactional sex (TS), and casual sex (CS). *Sex work* (SW) is defined as an explicit exchange of money (or gifts or other resources) for sex between a sex worker and client with upfront negotiation of the price of sex before any exchange takes place. TS is a more nuanced exchange of money, gifts, or other resources for sex between individuals with no prenegotiation of the price of sex (12, 13). CS occurs when individuals engage in sex but neither party expects to receive money, gifts, or other resources in return.

### Data Collected

#### Programmatic Mapping and Population Size Estimation

We modified the standard programmatic mapping method (14–21) for FSWs to also estimate the population size of young FSWs and other AGYW (14–24 years) who visited hotspots to meet partners for TS and CS (7). Mapping was conducted immediately prior to survey implementation, and the mapping findings were used to develop the sampling frame. For the purpose of mapping, Mombasa County and Dnipro were divided into existing administrative districts, which consisted of 9 and 23 data collection zones, respectively.

### Sample Size

Based on expected baseline values and odds ratios of HIV prevalence of the 3 study groups, with 80% statistical power and 95% confidence level, the sample sizes of the CS group, TS group, and SW group were 900, 450, and 450, respectively (Appendix A).

### Sampling Frame and Sampling Strategy

The population sizes of three groups were estimated for each hotspot and used to develop a sampling frame for each group. The hotspots served as the primary sampling units. The number of primary sampling units for each group was determined based on the required sample size and the mean number of target group members per hotspot. The hotspots were selected after stratification by typology and geographic distribution to ensure a representative sample of hotspots. Therefore, we selected 170, 85, and 85 hotspots in Mombasa and 174, 116, and 136 hotspots in Dnipro from the sampling frame, respectively, for the CS, TS, and SW groups. In each selected hotspot, the required sample size was proportional to the size of the estimated target population in the hotspot. Within each hotspot, outreach workers or mobilizers would randomly select participants to approach based on an agreed upon sampling strategy. Briefly, the mobilizer would visit the hotspot during busy times to survey the size and shape of the venue and the number of potential participants who might be in the space. Starting at different corners of the venue, the mobilizer was asked to draw an imaginary diagonal line across the venue. Any woman standing or seated along the imaginary line was approached for an interview. Depending on the expected sampling frame for the spot, every second or third woman might be approached. The same strategy was applied to selecting tables to approach within a venue. For public spaces, such as beaches or parks, mobilizers walked along the beach/park and selected whoever was thought to fall within the study criteria.

### Recruitment Strategy and Study Procedures

Tool development, data collection and data cleaning for *Transitions* were conducted in partnership with the International Centre for Reproductive Health Kenya (ICRH) in Mombasa [a non-governmental organization (NGO) providing HIV prevention services for sex workers] and with research partners Ukrainian Institute for Social Research after Oleksandr Yaremenko (UISR) in Kiev and DEFgroup in Dnipro and Virtus (an NGO providing HIV prevention services for sex workers) in Dnipro. The recruitment and study procedures, as detailed below, were adapted to the local context and designed to be consistent with methods used in previous studies in both sites and therefore varied slightly between the two sites.

#### Mombasa

Peer educators (former or current FSWs) from the FSW network of ICRH visited selected hotspots and prescreened individuals who were congregating at the hotspot for eligibility until the preallocated sample size for that hotspot was achieved. Interested potential participants were given an invitation card to come to an interview site. At the interview site, trained research assistants screened and enrolled the participants. Following written informed consent, a structured questionnaire was administered. If the participant consented to take part in the biological component of the study, she was introduced to a clinical officer or nurse counselor who performed HIV rapid testing with pretest and posttest counseling and collected additional biological samples. Study procedures were conducted in Swahili or English in Mombasa County. Data collection took place between April and November 2015.

#### Dnipro

Outreach workers (Virtus) and research assistants (DEFgroup) recruited participants and implemented the study under the direction of UISR. Virtus outreach workers visited and recruited participants from hotspots exclusively associated with SW such as brothels, apartments (small scale, private SW enterprises), and highways. If a potential participant met eligibility criteria and expressed interest in taking part in the study, she was escorted to a mobile clinic parked nearby where the Virtus outreach worker administered written informed consent and conducted the study procedures. Participants from hotspots not exclusive to SW were recruited by DEFgroup research assistants. For these participants, the written informed consent and interview were conducted on site or in a research van, and rapid HIV testing was conducted by a medical officer in an AIDS center outpatient clinic. Only participants who consented to the full study procedure (questionnaire and biological testing) were enrolled. Study procedures were conducted in Russian in Dnipro. Data collection took place between July and November 2015.

### Screening and Enrolment Numbers

**Figure 2:**
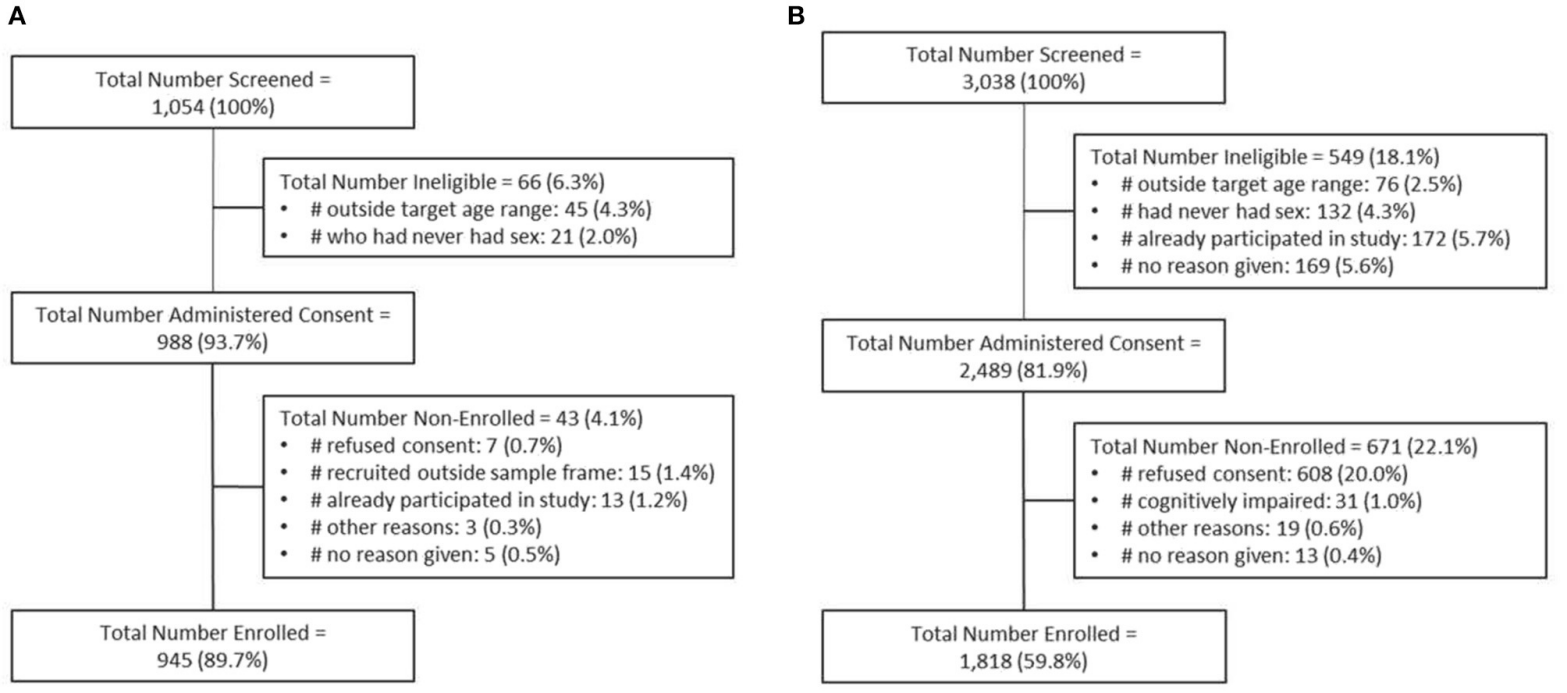
Schematic showing the numbers of participants screened, consented, and enrolled for *Transitions* in **(A)** Mombasa County, Kenya, and **(B)** Dnipro, Ukraine. Note: Data for screening and enrolment from Mombasa County are incomplete.

In Mombasa County, screening information was available on 1,054 women (Figure 2A). Of the 1,054 women screened, 988 (93.7%) eligible potential participants were invited to participate, and 945 (89.6%) were enrolled. We achieved 72.1% (*n* = 1,299) of the target sample size because of unanticipated delays in study commencement and participant recruitment. Among participants, 714, 177, and 408 women were categorized into the CS, TS, and SW groups, respectively.

In Dnipro, 3,038 women were screened, 2,489 eligible potential participants were invited to participate, and 1,818 (73.0%) were enrolled (Figure 2B). Among whom, 899, 466, and 453 women were categorized into the CS, TS, and SW groups, respectively.

### Questionnaire

The questionnaire collected information about participants' sociodemographic characteristics; timing of transition and access gap events (first sex, first exchange of sex for money or other resources, self-identification as sex workers, entry into formal SW, access to prevention program services); sexual behaviors (condom use, anal sex, sex under the influence of drugs or alcohol); partnerships (regular and first-time clients, regular and first-time TS partners, and husbands and boyfriends); alcohol use; injection and non-injection illicit drug use; experience of violence; access to HIV prevention and treatment program; testing for sexually transmitted and blood-borne infections and HIV; and reproductive health (pregnancies, abortions, contraceptives). The questionnaires were pilot tested in both sites prior to implementation.

**Figure 3:**
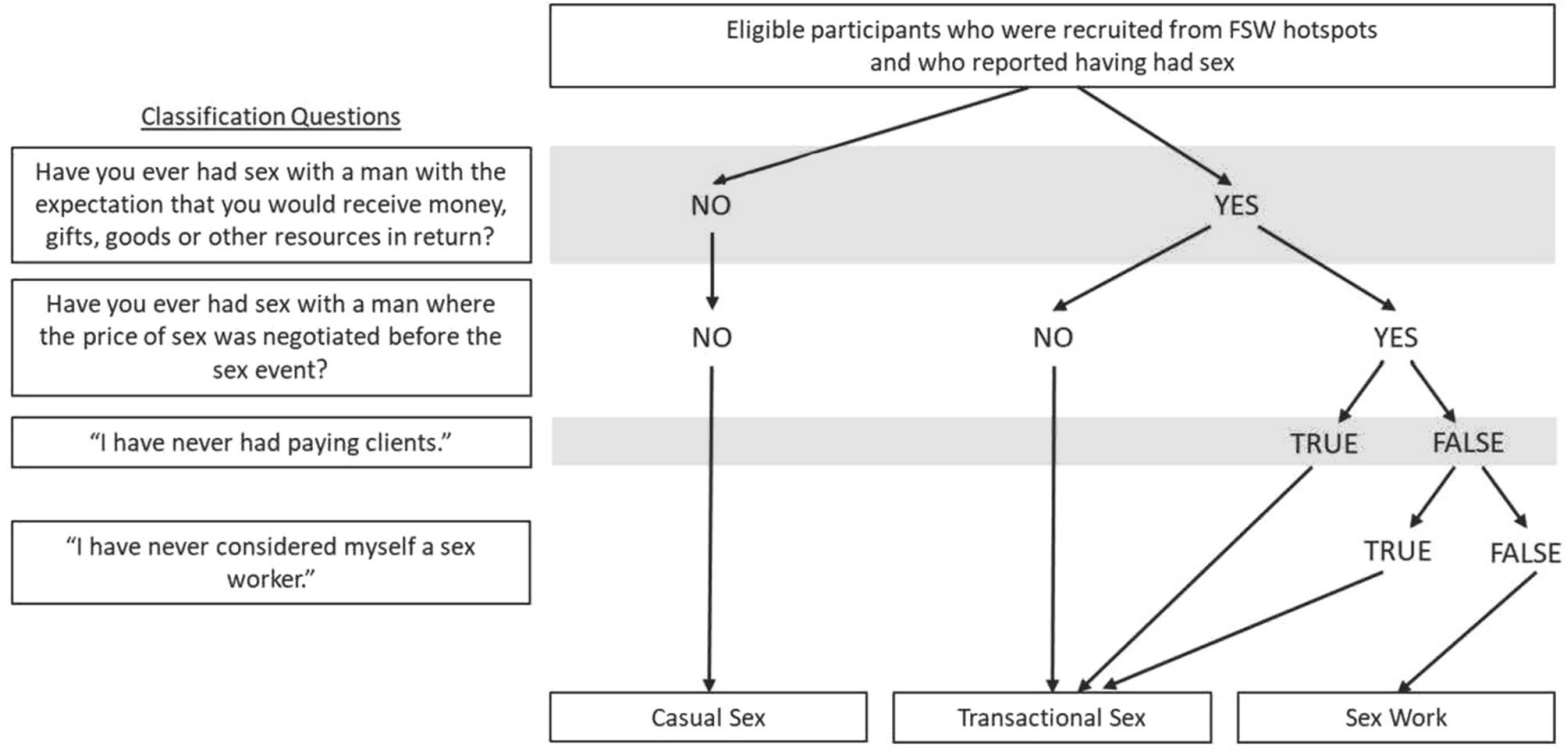
Algorithm for classifying participants into the three study groups in *Transitions*.

Figure 3 describes the algorithm used to categorize participants into 3 study groups (SW, TS, CS) based on their answers to a series of questions in the questionnaire: (1) “Have you ever had sex with a man with the expectation that you would receive money, gifts, goods, or other resources in return?”; and (2) “Have you ever had sex with a man where the price of sex was negotiated before the sex event?” If a participant answered “no” to both questions, she was directed to sections of the questionnaires for the CS group. If a participant answered “yes” to (1) but “no” to (2), she was directed to the TS group. If a participant answered “yes” to both (1) and (2), she was directed to the SW group. For individuals who answered “yes” to both criteria questions but later reported in subsequent questions not having ever had paying clients or not having ever had considered herself as a sex worker, she was redirected to the TS group (Figure 3).

Table 2 shows the sociodemographic characteristics of *Transitions* participants by study group.

**Table 2:** Sociodemographics of *Transitions* participants from (A) Mombasa County, Kenya, and (B) Dnipro, Ukraine.

**Kenya**		**All groups**		**Group 1**		**Group 2**		**Group 3**		***p***
				**Casual sex**		**Transactional sex**		**Sex work**		
		***n*** **=** **1,299**		***n*** **=** **714**		***n*** **=** **177**		***n*** **=** **408**		
		***n***	**%**	***n***	**%**	***n***	**%**	***n***	**%**	
**(A)**										
**Median age (IQR), years**		19 (17–21)		19 (17–21)		19 (18–21)		20 (18–22)		<0.0001
Education level	Never received formal education	24	1.85	13	1.82	3	1.69	8	1.96	0.0002
	Education level below secondary education	939	72.29	508	71.15	106	59.89	325	79.66	
	Full secondary education	294	22.63	173	24.23	57	32.2	64	15.69	
	Vocational education	6	0.46	1	0.14	2	1.13	3	15.69	
	Higher education, postgraduate	23	1.77	10	1.4	6	3.39	7	1.72	
	missing	13	1.00	9	1.26	3	1.69	1	0.25	
Currently a student	Yes	266	20.48	203	28.43	30	16.95	33	8.09	<0.0001
Marital status	Single, never married	1,206	92.84	668	93.56	164	92.66	374	91.67	0.0023
	Married, living with husband	11	0.85	8	1.12	2	1.13	1	0.25	
	Married, not living with husband	4	0.31	2	0.28	2	1.13	0	0	
	Not married, but live with a sex partner	30	2.31	21	2.94	2	1.13	7	1.72	
	Divorced/separated	45	3.46	14	1.96	6	3.39	25	6.13	
	Widowed	2	0.15	1	0.14	0	0	1	0.25	
	NA	1	0.08	0	0	1	0.56	0	0	
**Median age of first sex (IQR), years**		16 (14-17)		16 (15-18)		16 (18-21)		15 (14-17)		0.0002
Ever pregnant	Yes	493	37.95	189	26.47	70	39.55	234	57.35	<0.0001
**Dnipro, Ukraine**		**All groups**		**Group 1**		**Group 2**		**Group 3**		***p***
				**Casual sex**		**Transactional sex**		**Sex work**		
		***n*** **=** **1,818**		***n*** **=** **899**		***n*** **=** **469**		***n*** **=** **450**		
		***n***	**%**	***n***	**%**	***n***	**%**	***n***	**%**	
**(B)**										
**Median age (IQR), years**		20 (18–22)		19 (17–22)		21 (19–23)		20 (19–22)		<0.0001
Education level	Never received formal education	2	0.11	2	0.22	0	0	0	0	<0.0001
	Education level below secondary education	450	24.86	202	22.47	65	13.89	183	40.76	
	Full secondary education	238	13.09	90	10.01	64	13.65	84	18.67	
	Vocational education	802	44.11	421	46.83	217	46.27	164	36.44	
	Higher education, postgraduate	318	17.49	178	19.8	122	26.01	18	4.00	
	Missing	8	0.44	6	0.67	1	0.21	1	0.22	
Currently a student	Yes	782	43.01	520	57.84	200	42.64	62	13.78	<0.0001
Marital status	Single, never married	1,413	77.72	700	77.86	339	72.28	374	83.11	<0.0001
	Married, living with husband	116	6.38	84	9.34	24	5.12	8	1.78	
	Married, not living with husband	44	2.42	8	0.89	22	4.69	14	3.11	
	Not married, but live with a sex partner	192	10.56	92	10.23	69	14.71	31	6.89	
	Divorced/separated	44	2.42	14	1.56	11	2.35	19	4.22	
	Widowed	6	0.33	1	0.11	2	0.43	3	0.67	
	NA	3	0.17	0	0	2	0.43	1	0.22	
**Median age of first sex (IQR), years**		16 (15–17)		16 (15–17)		16 (15–17)		15 (14–16)		<0.0001
Ever pregnant	Yes	519	28.55	245	27.25	131	27.93	143	31.78	0.2093

### Biological Testing and Sample Collection

#### Mombasa

HIV testing followed the Kenya national testing algorithm using a series of 3 rapid tests on fingerprick blood samples (22). The first test was KHB HIV (1+2) Antibody (Colloidal Gold) Rapid Test (Shanghai Kehua Bio-engineering Co., Ltd, Shanghai, China); the second test was First Response HIV 1-2-O Rapid Whole Blood Test (Premier Medical Corporation Private Limited, Mumbai, India); and the tie-breaker test was Uni-Gold™ HIV Test (Trinity Biotech Plc, Bray, Ireland). If the first test was negative, the participant was informed of the negative results and was referred to the appropriate HIV prevention services. If the first test was positive, the second test was performed. If the second test was also positive, the participant was informed of the positive result and was referred to the appropriate HIV treatment services. If the second test was negative, the result was classified as discordant, and a third blood sample was collected for the tie-breaker test. A clinical officer/nurse counselor performed the tests along with pretest and posttest counseling and service referral in accordance with the Ministry of Health of Kenya (23). In *Transitions* Mombasa, 1,084 (83.4%) of 1,299 participants underwent HIV rapid testing.

Dried blood spot (DBS) specimens were collected from consenting participants for HIV and hepatitis C serological confirmation performed by the National HIV and Retrovirology Laboratories of the Public Health Agency of Canada (Winnipeg, Canada), using Avioq HIV-1 Microelisa System (Avioq Inc., Durham, NC, USA) and ORTHO® HCV Version 3.0 ELISA Test System (Ortho-Clinical Diagnostics, Inc., Raritan, NJ, USA). In *Transitions* Mombasa, 1,193 (91.8%) of 1,299 participants provided DBS specimens.

In addition, a self-collected urine sample, a self-collected vaginal secretion sample using a Softcup™ (menstrual hygiene disc) (Evofem Inc., San Diego, CA, USA), and a blood sample collected via venipuncture by the clinical officer/nurse counselor were also obtained from consenting participants. These biological samples will be testing for a selection of sexually transmitted and blood-borne organisms including *Ureaplasma urealyticum, Ureaplasma parvum, Neisseria gonorrhoeae, Chlamydia trachomatis, Mycoplasma genitalium, Mycoplasma hominis*, and *Trichomonas vaginalis* (Anyplex™ II STI-7 Detection Kit, Seegene, Seoul, Korea), herpes simplex virus (HerpeSelect® 2 ELISA IgG, Focus Diagnostics, Cypress, CA, USA), human papillomavirus (Cobas® HPV, Roche, Pleasanton, CA, USA), *Treponema pallidum*, and hepatitis B virus. Vaginal microbiome (via 16S rRNA sequencing) and cytokine profiles (Bio-Plex Pro™ Human Cytokine/Chemokine Panels, Bio-Rad, Hercules, CA, USA) were characterized in vaginal secretions. Phylogenetic analyses of HIV, hepatitis C virus, and human papillomavirus will be performed to understand viral genetic variation and evolution, geographic dispersal, and epidemic dynamics. These tests will be conducted in the University of Manitoba (Winnipeg, Canada) and the National HIV and Retrovirology Laboratories.

#### Dnipro

A combination rapid test for HIV, syphilis, and hepatitis B and C [New Vision Diagnostics Profitest Rapid Multi-Infectious Disease Test Card (HIV/HBsAg/hepatitis C virus/TP) (whole blood/serum/plasma) (InTec Products, Inc., Xiamen, China)] was used. Participants who tested positive for HIV on the rapid test were referred to the Dnipropetrovsk Oblast AIDS Center or Dnipro City AIDS Center for confirmatory laboratory testing. If HIV confirmatory laboratory testing was also positive, participants were referred to the appropriate treatment services within the AIDS Center. In *Transitions* Dnipro, 1,818 (100%) of 1,818 participants underwent HIV rapid testing.

### Statistical Methods

#### Epidemiological Analyses

**Figure 4:**
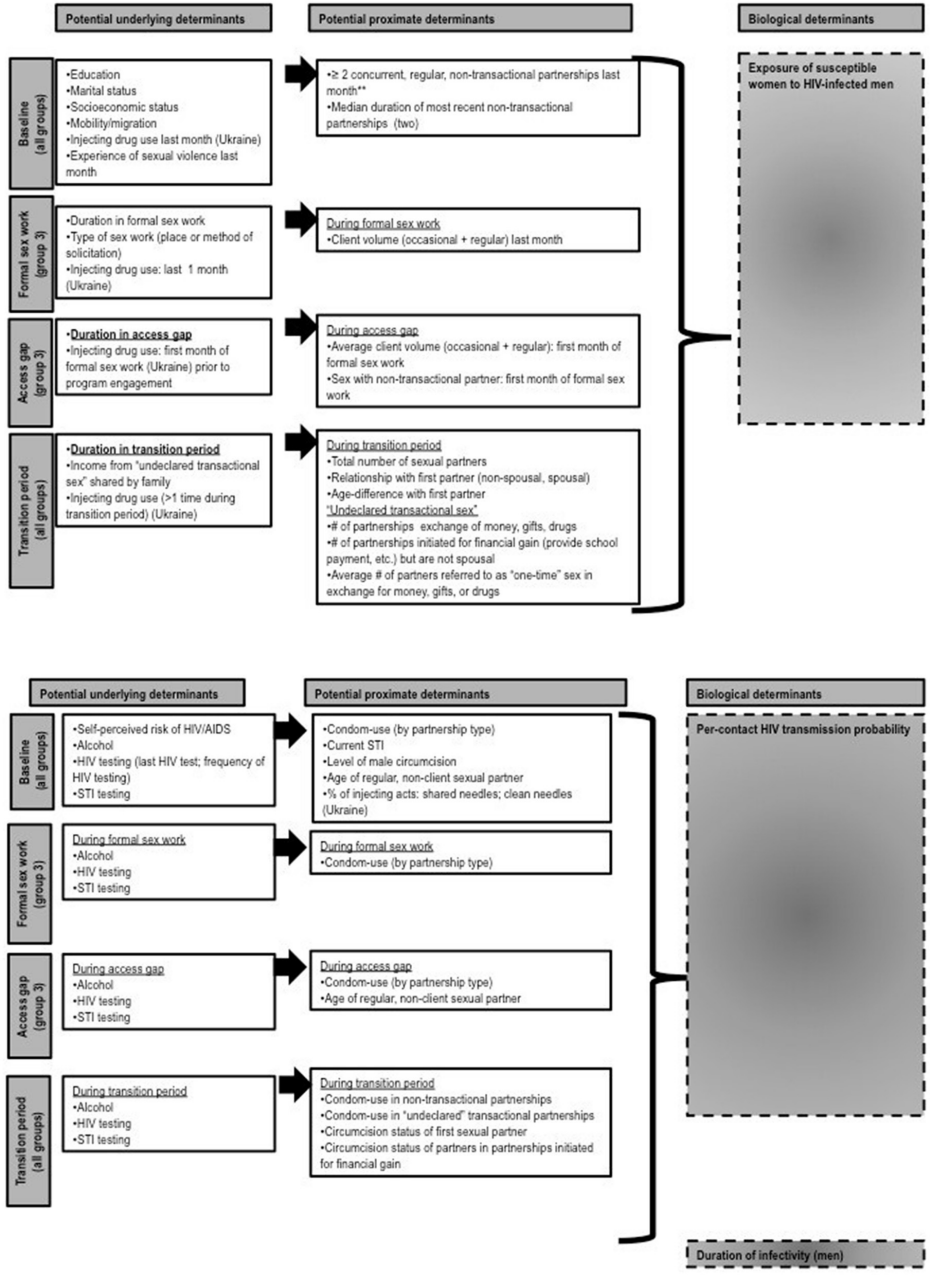
Proposed proximate determinant framework for objective 2. Biological determinants are unmeasured. Potential underlying and proximate determinants can be measured from a cross-sectional study and are grouped according to specific “periods” in respondent's sexual history: baseline (all groups), access gap, and the transition period. Note that the transition period for groups 1 and 2 refers to the entire duration of sexual activity (i.e., time from first sex) and is right-censured.

Descriptive analyses and multivariate logistics regression models using a proximate determinant framework (24) will be performed to address objectives 1 and 2 in each study site. We will build multivariate logistic regression models to explore potential variables, which could explain the observed differences between HIV prevalence and length of transition period or access gap in each study site. Potential explanatory variables are outlined in Figure 4 and are described under the proximate determinants framework. The proximate determinants (such as self-reported condom use or number of partners) are empirically measured and are markers for biological determinants (such as the HIV transmission probability per sex act or per partnership), which are unobserved, but ultimately lead to transmission (24). In the framework, underlying determinants act through the proximate determinants and ultimately through biological determinants (24). The framework can be used to explore risk factors associated with prevalent HIV infections, (22, 25) because it helps to disentangle and explore the influence of underlying determinants on HIV prevalence. While some elements will remain consistent, the framework will be modified for each study site according to the local epidemic context. The crude (univariate) and adjusted (multivariate) analyses will account for within-cluster homogeneity (due to the sampling design). Because sampling was conducted proportional to estimated size of hotspots (for each of the three groups), the samples are self-weighted and will not require sampling weights. We will perform multiple imputation for variables with >5% missing values as relevant for each analysis.

#### Transmission Dynamics Model Overview

We will use a setting-specific, compartmental (26), deterministic model of HIV transmission to estimate the following outcomes (objective 3): HIV incidence among FSWs, cumulative HIV infections in the total population that stem from infections acquired by women during the transition period and access gap (transmission population attributable fraction), and the transmission impact (infections averted) of expanding tailored interventions to the transition period and access gap.

#### Transmission Model Structure, Parameters, and Key Assumptions

**Figure 5:**
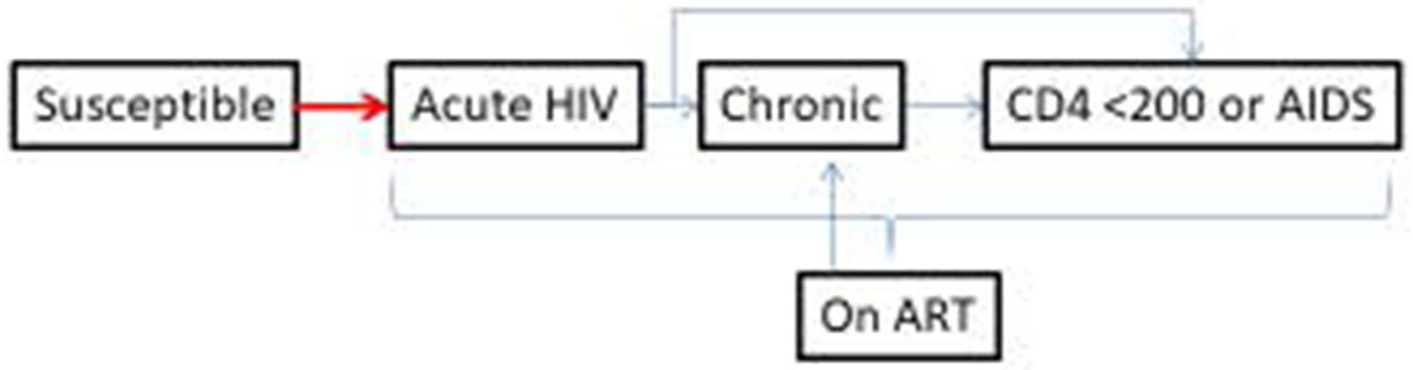
Mathematical model: HIV-related biology.

**Figure 6:**
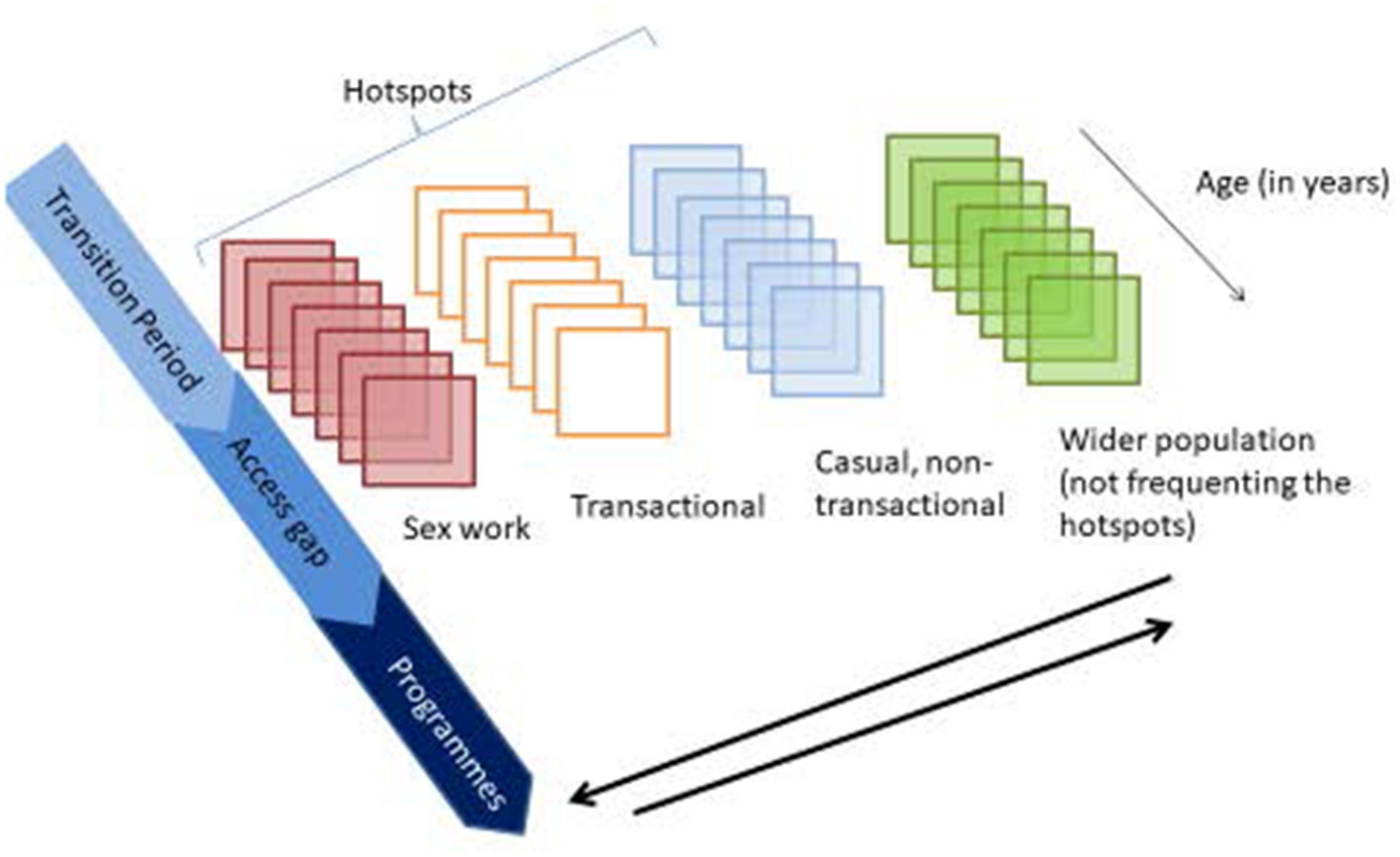
Mathematical model: demographic and sexual partnerships.

We will report and conduct the modeling in accordance with recommended best practices in use and justification of parameters based on the relevant research question (27) and as commonly used in transmission models that include SW (28–31). The model structure and therefore parameters (data inputs) include biological states that reflect a simplified natural history of infection and health care (susceptible, acute HIV infection, chronic HIV infection, and antiretroviral treatment). The demographic and risk strata are more complex in order to address the research questions and include FSWs, women who socialize at SW hotspots and are engaged in TS and CS (non-transactional); and women in the wider population (who may also be engaged in TS and CS). The age structure is divided as follows: females from ages 14 to 24 years are stratified into 1-year bands and then one strata for women over 24 years of age; males are stratified into two categories (<24 and >24 years of age). FSWs will be divided according to their time since starting TS, with a structure representing division of the life history of sex workers into relevant distinct periods, such as the transition period, parameterized with durations obtained from the proposed study for each site (Figures 5, 6). Individuals transition from susceptible to acute HIV infection, based on a force of infection (risk per susceptible) that is dependent on number of sexual partnerships by type of partnership, number and type of sex acts within each partnership type, and which types of partnerships occur between each subgroup (i.e., who has sex with whom, or the sexual mixing pattern). Sexual partnership types include main (long-term), CS, TS, and paid sex. HIV transmission is simulated per sex act, and condom use and efficacy will be modeled per sex act, whereas use of HIV pre-exposure prophylaxis (which can reduce HIV susceptibility as drawn from literature on individual-level effectiveness) (32, 33) will be modeled by coverage per subgroup. HIV pre-exposure prophylaxis was not part of the HIV prevention program in 2015 when the survey was conducted, but will be included for projected intervention analyses for projected impact of expanding tailored interventions.

The key model assumption (because of limitations of a cross-sectional data source) is that the relative and absolute differences in sex acts, partnership types, condom use, etc., across subgroups of women aged 14 to 24 years are stable over time. Other key model assumptions include (a) instantaneous partnership formation and dissolution (i.e., duration of partnerships is not explicitly captured); (b) partnerships and risks do not vary after age 24 years (i.e., relative stability in types of partnerships and risks after age 24 years); and (c) homogeneity in risks of transmission by health care states beyond on-treatment versus untreated. Finally, the model assumes that the trajectory of health care may vary between subgroups, but features such as reinitiation of HIV treatment are not dependent on how long one was on treatment (i.e., it is a memory-less system).

### Data Sources and Inputs for Model Parameterization and Fitting

The data inputs (parameters) from the empirical study comprise subgroup population size, transition rates between compartments, sexual partnerships, and who has sex with whom patterns (sexual mixing). Biological data inputs such as probability of transmission per sex act and by type of sex act will be drawn from the literature.

In addition to the empirical data collected as part of the proposed study, the following individual-level data [previously collected by our team and securely stored at the Centre for Global Public Health] will be used for model parameterization: behavioral and biological data from serial cross-sectional and cohort studies of formal FSWs;(34–36) serial general population surveys; up-to-date mapping and enumeration studies;(36, 37) polling booth surveys of formal FSWs;(38) local program data;(39) and publicly available aggregate data, such as Demographic and Health Surveys (9, 40, 41) and census, baseline mortality, and population growth data (42). The natural history of HIV and transmission probabilities (in the absence and presence of antiretroviral treatment) will be obtained from the literature (43–45).

#### Validating the Model Output Against Empirical Data (Model Calibration or Fitting)

Each site will be parameterized separately and fitted within a Bayesian framework (28, 29, 46) to the following region-specific epidemiological data up to and including 2019: (i) empirical estimates of prevalent HIV infections by length of transition period/access gap, data collected from the study; (ii) trends in HIV prevalence among AGYW overall, among FSWs, among clients, and among the wider population of males and females. We will sample parameters using Latin hypercube sampling and obtain multiple fits (“epidemic realizations”) with which to perform the dynamical model analyses (28). This fitting procedure will allow us to take uncertainty in parameter values into account, so that the simulated outputs reflect the range of epidemics consistent with population-specific behavioral characteristics and HIV prevalence data (30).

#### Dynamical Model Analysis

Once epidemic trajectories (“fits”) consistent with the observed epidemiological data are found, simulated counterfactuals will be developed, where all parameters are kept constant except for the variable of interest. In the case of objective 3, we will model increasing intervention access to women during the transition period and access gap. Using mathematical modeling and a simulated counterfactual, we will obtain a direct estimate of the marginal benefit of expanding tailored programs in this way over the subsequence 1, 5, and 10 years.

A preliminary version of the dynamic model has been developed and has undergone model checks with preliminary analyses conducted under assumptions of an endemic equilibrium state (31). The model is written in MATLAB (version 2018a).

## Anticipated Results

The proximate determinants framework of HIV transmission provides a conceptual basis for examining underlying social, contextual, and demographic determinants, as well as individual proximate and biological determinants predicting risk of HIV infection (24). We will use and compare univariate and adjusted logistic regression to examine the magnitude and direction of an independent relationship between observed differences between HIV prevalence (outcome) and length of transition period or access gap (exposure variables) in each study site (47). Adjusted regression models will include age and also account for a priori potential confounders identified in prior literature to be associated with prevalent HIV infection among AGYW and among FSWs in Kenya. This study will generate novel findings on the length of the transition period and access gap. This study will also deepen our understanding about the HIV risks, vulnerabilities, and prevalence among AGYW during the transition period and how these risks vary across the two study contexts.

We will use the mathematical model to perform the following specific analyses:

1. Estimate the current and projected HIV incidence among women during the transition period and access gap for each study site.
2. Estimate the potential impact of tailored interventions and baseline general population interventions on HIV incidence, HIV prevalence, and the fraction of HIV infections averted in FSWs and clients (and the wider community) under the following scenarios:
  a) Base-case scenario: existing tailored interventions reach FSWs after the transition period and access gap.
  b) Early tailored interventions scenario: existing tailored interventions reach women earlier in their formal SW career, i.e., shorten the access gap (by 25%, 50%, 75%, and 100%).
  c) Expanded tailored interventions scenario (“feasible”): expand tailored interventions to reach women during the transition period. This scenario will be informed by program implementers and knowledge users on our team to simulate “feasible” strategies and achievable levels of “coverage” of women during the transition period.
  d) Early and expanded tailored interventions (“optimistic”): expand tailored interventions to reach women during the transition period, at levels of coverage achieved by existing tailored interventions with self-identified FSWs.

Intervention tools will include increased condom use and a simplified representation of combination antiretroviral treatment. For each specific analysis described above, the median and uncertainty bounds (95% credible intervals) will be reported, and an uncertainty analysis will be performed to determine which input parameters were most influential.

## Discussion

The *Transitions* data resource includes a unique study population and hotspot-based recruitment strategy that enables the examination of early HIV risk and vulnerabilities among AGYW who engaged in formal SW and other partnership types, including TS and CS. To date, there are only a few studies on young FSWs (31, 48–50) and fewer yet that investigate the overlapping risks between TS and formal SW (51). *Transitions* has the potential to generate new evidence to inform the HIV prevention, sexual and reproductive health, and other related health needs of a subpopulation of “high-risk” young women not currently covered by FSW programs.

### Limitations

The cross-sectional nature of the study means association can be inferred but not causality. Cohort effects may also influence measures compared across age groups and thus should be considered. Another limitation is that Mombasa participants were able to opt out of the biological testing component, which may introduce a selection bias and will need to be accounted for in future analyses. Face-to-face interviews could introduce social desirability biases in participants' response due to the sensitive nature of some of the information elicited by the questionnaire (52, 53). Finally, we are using a cross-sectional survey to examine a life-course measure (transition period and access gap) and dynamic modeling to estimate the role of the transition period and the access gap. The gold standard to measure incidence and onward transmission during these periods of time would be a prospective cohort study (in the absence of any HIV interventions) or a community randomized controlled trial (where the “control” community provides our outcome of interest). However, both approaches are prone to intervention bias because it would be unethical to withhold behavioral interventions (such as condom promotion), treatment for bacterial sexually transmitted infections, and HIV testing—interventions that are being provided by the existing tailored interventions for self-identified FSWs who are already registered in local HIV prevention programs. Thus, we are restricted to indirectly estimating the characteristics of the transition period and access gap and indirectly estimating HIV incidence during the transition period and access gaps and their contribution to onward transmission. The statistical and dynamical models are thus limited by the following: (1) measurement error due to recall bias (especially with respect to duration of the transition period and access gap) and (2) cohort effects (changes over time, even after adjusting for age). Additional limitations of the transmission dynamics modeling include additional simplifying assumptions surrounding sexual life course (because of the cross-sectional nature of the survey, which will lead to right censoring) and sexual mixing patterns (because our parameters are restricted to self-reported data from the respondents and not their sexual partners).

## Conclusion

Taken together, this study design and implementation provide comprehensive behavioral, structural, and biological data on a high-risk group of adolescent girls and young women in two distinct sociocultural and epidemiologic contexts. All study findings will be shared through publications in peer-reviewed scientific journals, and via webinars, evidence briefs, written reports, and knowledge-exchange events with local government and non-governmental partners, including community-based organizations and FSW communities in each region.

## Data Availability Statement

The raw data supporting the conclusions of this article will be made available by the authors, without undue reservation.

## Ethics Statement

The studies involving human participants were reviewed and approved by the Human Research Ethics Board at the University of Manitoba in Canada (HS16557 [H2013:295]), the Ethical Review Committee Board at the Sociological Association of Ukraine, Committee on Medical Ethics of the L. Gromashevsky Institute of Epidemiology and Infectious Diseases at the National Academy of Medical Sciences of Ukraine; and the Kenyatta National Hospital/University of Nairobi Ethics and Research Committee (P497/10/2013), and the Research Permit Committee of the National Commission for Science, Technology and Innovation, in Kenya. Export of all collected biological samples from Kenya was granted by the Office of the Director of Medical Service of the Government of Kenya Ministry of Health. Written informed consent from the participants' legal guardian/next of kin was not required to participate in this study in accordance with the national legislation and the institutional requirements.

## Author Contributions

MB and SMi conceptualized and designed the study. EC and MB prepared the first draft of the manuscript. HMa conducted the data cleaning and analyses with input from SMi and MB. JB was involved in the conceptualization of the study and study design. PB, HMu, and PG oversaw the field-work and data collection in Kenya. OB and DP oversaw the field-work and data collection in Ukraine. EC was the research coordinator for the study, supported tool development and was involved in mapping, enumeration, and data collection. SI led the mapping, enumeration, and was involved in data collection and analyses. SMo and RL provided input on study design and tool development and questionnaire items. MP and LL were involved in manuscript editing. PS oversaw the laboratory diagnostics. All authors were involved in interpretation of results, critically reviewed, and provided final approval for the manuscript.

## Conflict of Interest

The authors declare that the research was conducted in the absence of any commercial or financial relationships that could be construed as a potential conflict of interest.

## Footnotes

^1^UNAIDS AIDSinfo. Available online at: http://aidsinfo.unaids.org/.

## Abbreviations

AGYW: adolescent girls and young women
CS: casual sex
DBS: dried blood spot
FSW: female sex worker
ICRH: International Centre for Reproductive Health Kenya
SW: sex work
TS: transactional sex
UISR: Ukrainian Institute for Social Research after Oleksandr Yaremenko.
