# Supplementary material for: Transitions: Novel Study Methods to Understand Early HIV Risk Among Adolescent Girls and Young Women in Mombasa, Kenya, and Dnipro, Ukraine
Source: Front Reprod Health. 2020 Sep 10;2:7. doi: 10.3389/frph.2020.00007 (PMC9580775; doi:10.3389/frph.2020.00007)
Supplement: Supplementary file 1 [file Table_1.DOCX]

**Appendix A: Sample Size Calculations**

Group 1: Women aged 14-24 who have had their first sex, but not engaged in transactional sex or sex work

Group 2: Women aged 15-19 years, who engage in transactional sex

Group 3: Women aged 15-19 years, who engage in commercial sex

Sample sizes for each group included in this survey have been calculated on the basis of the following factors typically used in surveys with probability samples:

1. The expected value of key indicators: HIV prevalence;
2. The odds ratio of HIV prevalence compared to reference group (Group 1)
3. Confidence level;
4. Statistical power.

The sample size for each study group has been derived using the HIV prevalence level below, with a power of 80%, 95% confidence level. The sample size is computed using the following equation:

n = (Zβ+Zα/2)^2^* p (1-p) (r+1)

d*^2 * r

OR = 4 for Group 1 vs. Group 2 (p1/p0); OR = 2 for Group 2 vs. Group 3 (p1/p0)

d* = p1-p0; p = (p1+rp0)/(1+r)

| p1 = | the estimated proportion of the Group 2 |
| --- | --- |
| p0 = | the estimated proportion of the Group 1 |
| Z1-α = | the z-score corresponding to the probability with which it is desired to be able to conclude that an observed change of size (P2 - P1) would not have occurred by chance |
| Z1-β = | the z-score corresponding to the degree of confidence with which it is desired to be certain of detecting a change of size (P2 - P1), if as change actually occurred |

r = ratio of number of unexposed group to exposed group

The following assumptions have been made regarding these parameters:

1. Expected baseline value: 0.7% for Group 1, 5-6% for Group 2 and 12-16% for Group 3.
2. Odds ratio of HIV prevalence: 4 and 2. This refers to the odds ratio of HIV prevalence among the Group 2 compared to Group 1, and Group 3 compared to Group 2.
3. The alpha level has been set at 0.05, corresponding to 95% confidence in the observed estimates.
4. The beta level has been set at 0.20, corresponding to 80% power.
5. The ratio of unexposed to exposed (r)= 1:3 for Group 1 and Group 2; 1:2 for Group 2 and Group 3.
6. Design effect: 2 for cluster sampling. This adjusts for the use of sampling designs that are not simple random methods, e.g. cluster sampling.

Minimum sample size requirements per target group per study unit to measure differences between groups at the levels of significance and power indicated above are summarized in the table below.

| Survey Population | Indicator | Expected baseline value | OR | % in denominator | Design  Effect | Required sample size | Sample size (rounded off) |
| --- | --- | --- | --- | --- | --- | --- | --- |
| Group 1 | HIV prevalence | 0.7% | -- | ALL | 2 | 989 | 900 |
| Group 2 | HIV prevalence | 5-6% | D1/D2: 4 | ALL | 2 | 452 | 450 |
| Group 3 | HIV prevalence | 10-15% | D2/D3:  2 | ALL | 2 | 452 | 450 |
